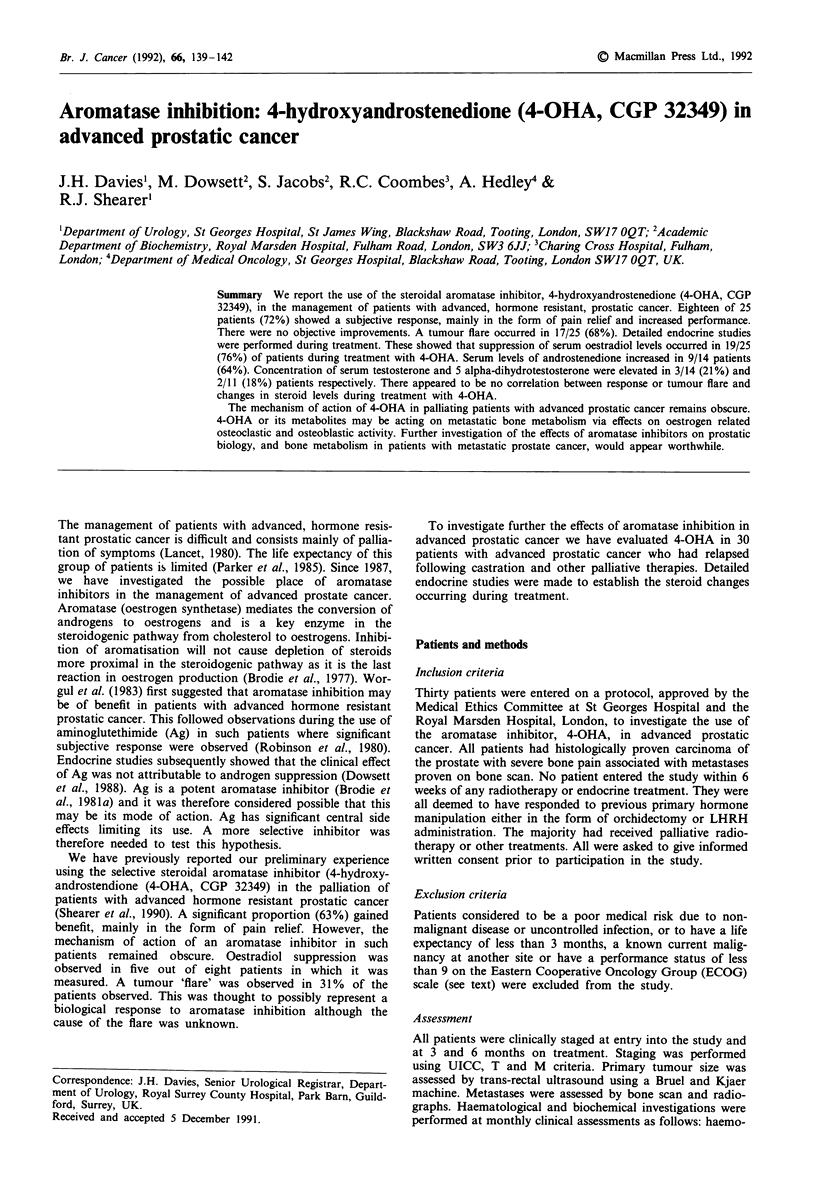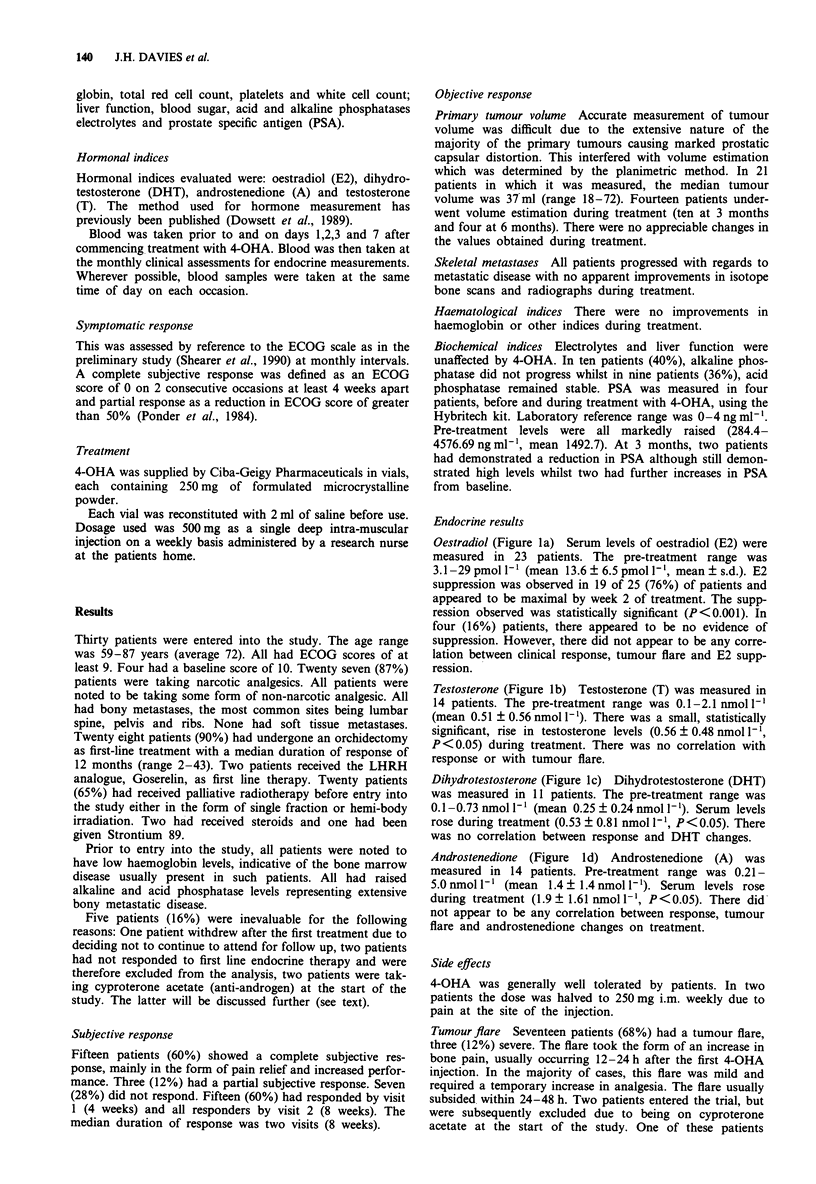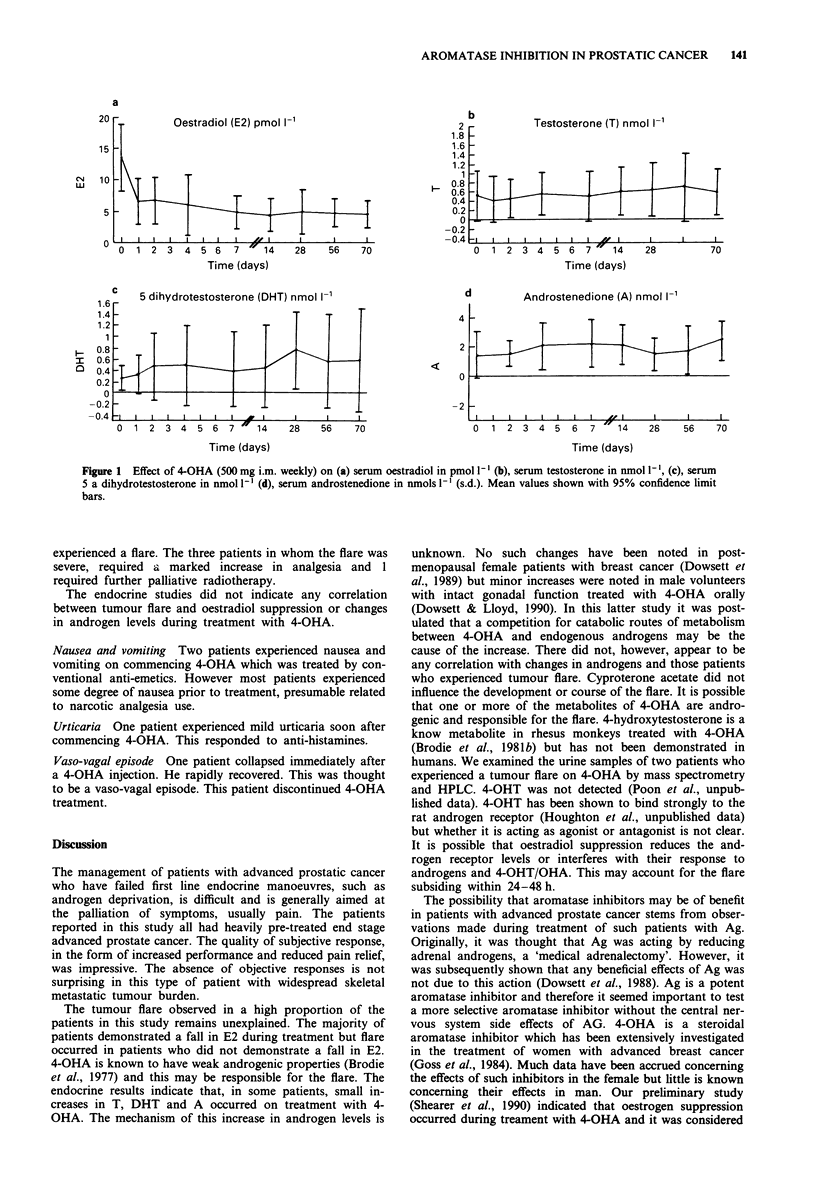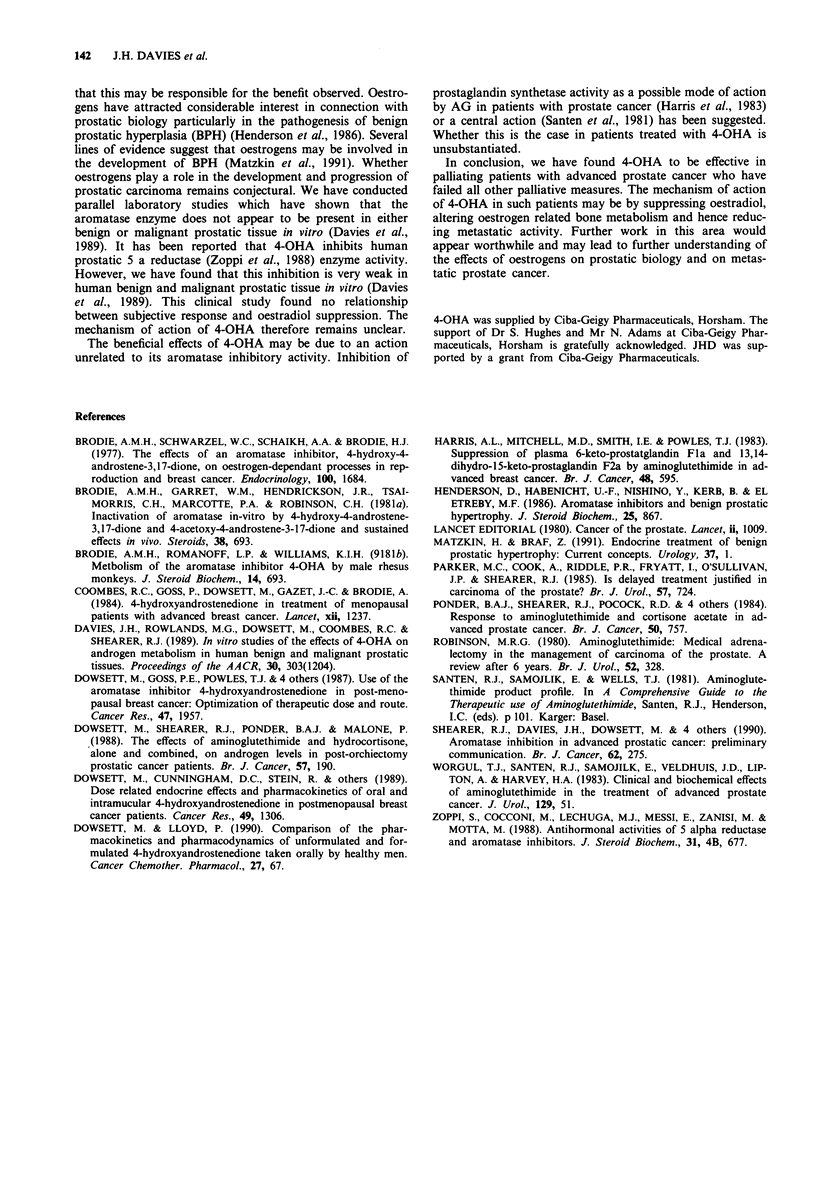# Aromatase inhibition: 4-hydroxyandrostenedione (4-OHA, CGP 32349) in advanced prostatic cancer.

**DOI:** 10.1038/bjc.1992.231

**Published:** 1992-07

**Authors:** J. H. Davies, M. Dowsett, S. Jacobs, R. C. Coombes, A. Hedley, R. J. Shearer

**Affiliations:** Department of Urology, St Georges Hospital, Tooting, London, UK.

## Abstract

We report the use of the steroidal aromatase inhibitor, 4-hydroxyandrostenedione (4-OHA, CGP 32349), in the management of patients with advanced, hormone resistant, prostatic cancer. Eighteen of 25 patients (72%) showed a subjective response, mainly in the form of pain relief and increased performance. There were no objective improvements. A tumour flare occurred in 17/25 (68%). Detailed endocrine studies were performed during treatment. These showed that suppression of serum oestradiol levels occurred in 19/25 (76%) of patients during treatment with 4-OHA. Serum levels of androstenedione increased in 9/14 patients (64%). Concentration of serum testosterone and 5 alpha-dihydrotestosterone were elevated in 3/14 (21%) and 2/11 (18%) patients respectively. There appeared to be no correlation between response or tumour flare and changes in steroid levels during treatment with 4-OHA. The mechanism of action of 4-OHA in palliating patients with advanced prostatic cancer remains obscure. 4-OHA or its metabolites may be acting on metastatic bone metabolism via effects on oestrogen related osteoclastic and osteoblastic activity. Further investigation of the effects of aromatase inhibitors on prostatic biology, and bone metabolism in patients with metastatic prostate cancer, would appear worthwhile.


Br. J. Cancer (1992), 66, 139–142 © Macmillan Press Ltd., 1992

### Aromatase inhibition: 4-hydroxyandrostenedione (4-OHA, CGP 32349) in advanced prostatic cancer

J.H. Davies[1], M. Dowsett[2], S. Jacobs[2], R.C. Coombes[3], A. Hedley[4] & R.J. Shearer[1]

[1]*Department of Urology, St Georges Hospital, St James Wing, Blackshaw Road, Tooting, London, SW17 0QT;* [2]*Academic Department of Biochemistry, Royal Marsden Hospital, Fulham Road, London, SW3 6JJ;* [3]*Charing Cross Hospital, Fulham, London;* [4]*Department of Medical Oncology, St Georges Hospital, Blackshaw Road, Tooting, London SW17 0QT, UK.*

**Summary** We report the use of the steroidal aromatase inhibitor, 4-hydroxyandrostenedione (4-OHA, CGP 32349), in the management of patients with advanced, hormone resistant, prostatic cancer. Eighteen of 25 patients (72%) showed a subjective response, mainly in the form of pain relief and increased performance. There were no objective improvements. A tumour flare occurred in 17/25 (68%). Detailed endocrine studies were performed during treatment. These showed that suppression of serum oestradiol levels occurred in 19/25 (76%) of patients during treatment with 4-OHA. Serum levels of androstenedione increased in 9/14 patients (64%). Concentration of serum testosterone and 5 alpha-dihydrotestosterone were elevated in 3/14 (21%) and 2/11 (18%) patients respectively. There appeared to be no correlation between response or tumour flare and changes in steroid levels during treatment with 4-OHA.

The mechanism of action of 4-OHA in palliating patients with advanced prostatic cancer remains obscure. 4-OHA or its metabolites may be acting on metastatic bone metabolism via effects on oestrogen related osteoclastic and osteoblastic activity. Further investigation of the effects of aromatase inhibitors on prostatic biology, and bone metabolism in patients with metastatic prostate cancer, would appear worthwhile.

The management of patients with advanced, hormone resistant prostatic cancer is difficult and consists mainly of palliation of symptoms (Lancet, 1980). The life expectancy of this group of patients is limited (Parker *et al.*, 1985). Since 1987, we have investigated the possible place of aromatase inhibitors in the management of advanced prostate cancer. Aromatase (oestrogen synthetase) mediates the conversion of androgens to oestrogens and is a key enzyme in the steroidogenic pathway from cholesterol to oestrogens. Inhibition of aromatisation will not cause depletion of steroids more proximal in the steroidogenic pathway as it is the last reaction in oestrogen production (Brodie *et al.*, 1977). Worgul *et al.* (1983) first suggested that aromatase inhibition may be of benefit in patients with advanced hormone resistant prostatic cancer. This followed observations during the use of aminoglutethimide (Ag) in such patients where significant subjective response were observed (Robinson *et al.*, 1980). Endocrine studies subsequently showed that the clinical effect of Ag was not attributable to androgen suppression (Dowsett *et al.*, 1988). Ag is a potent aromatase inhibitor (Brodie *et al.*, 1981*a*) and it was therefore considered possible that this may be its mode of action. Ag has significant central side effects limiting its use. A more selective inhibitor was therefore needed to test this hypothesis.

We have previously reported our preliminary experience using the selective steroidal aromatase inhibitor (4-hydroxyandrostendione (4-OHA, CGP 32349) in the palliation of patients with advanced hormone resistant prostatic cancer (Shearer *et al.*, 1990). A significant proportion (63%) gained benefit, mainly in the form of pain relief. However, the mechanism of action of an aromatase inhibitor in such patients remained obscure. Oestradiol suppression was observed in five out of eight patients in which it was measured. A tumour 'flare' was observed in 31% of the patients observed. This was thought to possibly represent a biological response to aromatase inhibition although the cause of the flare was unknown.

Correspondence: J.H. Davies, Senior Urological Registrar, Department of Urology, Royal Surrey County Hospital, Park Barn, Guildford, Surrey, UK.

Received and accepted 5 December 1991.

To investigate further the effects of aromatase inhibition in advanced prostatic cancer we have evaluated 4-OHA in 30 patients with advanced prostatic cancer who had relapsed following castration and other palliative therapies. Detailed endocrine studies were made to establish the steroid changes occurring during treatment.

### Patients and methods

### *Inclusion criteria*

Thirty patients were entered on a protocol, approved by the Medical Ethics Committee at St Georges Hospital and the Royal Marsden Hospital, London, to investigate the use of the aromatase inhibitor, 4-OHA, in advanced prostatic cancer. All patients had histologically proven carcinoma of the prostate with severe bone pain associated with metastases proven on bone scan. No patient entered the study within 6 weeks of any radiotherapy or endocrine treatment. They were all deemed to have responded to previous primary hormone manipulation either in the form of orchidectomy or LHRH administration. The majority had received palliative radiotherapy or other treatments. All were asked to give informed written consent prior to participation in the study.

### *Exclusion criteria*

Patients considered to be a poor medical risk due to non-malignant disease or uncontrolled infection, or to have a life expectancy of less than 3 months, a known current malignancy at another site or have a performance status of less than 9 on the Eastern Cooperative Oncology Group (ECOG) scale (see text) were excluded from the study.

### *Assessment*

All patients were clinically staged at entry into the study and at 3 and 6 months on treatment. Staging was performed using UICC, T and M criteria. Primary tumour size was assessed by trans-rectal ultrasound using a Bruel and Kjaer machine. Metastases were assessed by bone scan and radiographs. Haematological and biochemical investigations were performed at monthly clinical assessments as follows: haemoglobin, total red cell count, platelets and white cell count; liver function, blood sugar, acid and alkaline phosphatases electrolytes and prostate specific antigen (PSA).

*Hormonal indices*

Hormonal indices evaluated were: oestradiol (E2), dihydrotestosterone (DHT), androstenedione (A) and testosterone (T). The method used for hormone measurement has previously been published (Dowsett *et al.*, 1989).

Blood was taken prior to and on days 1,2,3 and 7 after commencing treatment with 4-OHA. Blood was then taken at the monthly clinical assessments for endocrine measurements. Wherever possible, blood samples were taken at the same time of day on each occasion.

*Symptomatic response*

This was assessed by reference to the ECOG scale as in the preliminary study (Shearer *et al.*, 1990) at monthly intervals. A complete subjective response was defined as an ECOG score of 0 on 2 consecutive occasions at least 4 weeks apart and partial response as a reduction in ECOG score of greater than 50% (Ponder *et al.*, 1984).

*Treatment*

4-OHA was supplied by Ciba-Geigy Pharmaceuticals in vials, each containing 250 mg of formulated microcrystalline powder.

Each vial was reconstituted with 2 ml of saline before use. Dosage used was 500 mg as a single deep intra-muscular injection on a weekly basis administered by a research nurse at the patients home.

### Results

Thirty patients were entered into the study. The age range was 59–87 years (average 72). All had ECOG scores of at least 9. Four had a baseline score of 10. Twenty seven (87%) patients were taking narcotic analgesics. All patients were noted to be taking some form of non-narcotic analgesic. All had bony metastases, the most common sites being lumbar spine, pelvis and ribs. None had soft tissue metastases. Twenty eight patients (90%) had undergone an orchidectomy as first-line treatment with a median duration of response of 12 months (range 2–43). Two patients received the LHRH analogue, Goserelin, as first line therapy. Twenty patients (65%) had received palliative radiotherapy before entry into the study either in the form of single fraction or hemi-body irradiation. Two had received steroids and one had been given Strontium 89.

Prior to entry into the study, all patients were noted to have low haemoglobin levels, indicative of the bone marrow disease usually present in such patients. All had raised alkaline and acid phosphatase levels representing extensive bony metastatic disease.

Five patients (16%) were inevaluable for the following reasons: One patient withdrew after the first treatment due to deciding not to continue to attend for follow up, two patients had not responded to first line endocrine therapy and were therefore excluded from the analysis, two patients were taking cyproterone acetate (anti-androgen) at the start of the study. The latter will be discussed further (see text).

*Subjective response*

Fifteen patients (60%) showed a complete subjective response, mainly in the form of pain relief and increased performance. Three (12%) had a partial subjective response. Seven (28%) did not respond. Fifteen (60%) had responded by visit 1 (4 weeks) and all responders by visit 2 (8 weeks). The median duration of response was two visits (8 weeks).

*Objective response*

*Primary tumour volume* Accurate measurement of tumour volume was difficult due to the extensive nature of the majority of the primary tumours causing marked prostatic capsular distortion. This interfered with volume estimation which was determined by the planimetric method. In 21 patients in which it was measured, the median tumour volume was 37 ml (range 18–72). Fourteen patients underwent volume estimation during treatment (ten at 3 months and four at 6 months). There were no appreciable changes in the values obtained during treatment.

*Skeletal metastases* All patients progressed with regards to metastatic disease with no apparent improvements in isotope bone scans and radiographs during treatment.

*Haematological indices* There were no improvements in haemoglobin or other indices during treatment.

*Biochemical indices* Electrolytes and liver function were unaffected by 4-OHA. In ten patients (40%), alkaline phosphatase did not progress whilst in nine patients (36%), acid phosphatase remained stable. PSA was measured in four patients, before and during treatment with 4-OHA, using the Hybritech kit. Laboratory reference range was 0–4 ng $ml^{-1}$. Pre-treatment levels were all markedly raised (284.4–4576.69 ng $ml^{-1}$, mean 1492.7). At 3 months, two patients had demonstrated a reduction in PSA although still demonstrated high levels whilst two had further increases in PSA from baseline.

*Endocrine results*

*Oestradiol* (Figure 1a) Serum levels of oestradiol (E2) were measured in 23 patients. The pre-treatment range was 3.1–29 pmol $l^{-1}$ (mean 13.6 ± 6.5 pmol $l^{-1}$, mean ± s.d.). E2 suppression was observed in 19 of 25 (76%) of patients and appeared to be maximal by week 2 of treatment. The suppression observed was statistically significant ($P < 0.001$). In four (16%) patients, there appeared to be no evidence of suppression. However, there did not appear to be any correlation between clinical response, tumour flare and E2 suppression.

*Testosterone* (Figure 1b) Testosterone (T) was measured in 14 patients. The pre-treatment range was 0.1–2.1 nmol $l^{-1}$ (mean 0.51 ± 0.56 nmol $l^{-1}$). There was a small, statistically significant, rise in testosterone levels (0.56 ± 0.48 nmol $l^{-1}$, $P < 0.05$) during treatment. There was no correlation with response or with tumour flare.

*Dihydrotestosterone* (Figure 1c) Dihydrotestosterone (DHT) was measured in 11 patients. The pre-treatment range was 0.1–0.73 nmol $l^{-1}$ (mean 0.25 ± 0.24 nmol $l^{-1}$). Serum levels rose during treatment (0.53 ± 0.81 nmol $l^{-1}$, $P < 0.05$). There was no correlation between response and DHT changes.

*Androstenedione* (Figure 1d) Androstenedione (A) was measured in 14 patients. Pre-treatment range was 0.21–5.0 nmol $l^{-1}$ (mean 1.4 ± 1.4 nmol $l^{-1}$). Serum levels rose during treatment (1.9 ± 1.61 nmol $l^{-1}$, $P < 0.05$). There did not appear to be any correlation between response, tumour flare and androstenedione changes on treatment.

*Side effects*

4-OHA was generally well tolerated by patients. In two patients the dose was halved to 250 mg i.m. weekly due to pain at the site of the injection.

*Tumour flare* Seventeen patients (68%) had a tumour flare, three (12%) severe. The flare took the form of an increase in bone pain, usually occurring 12–24 h after the first 4-OHA injection. In the majority of cases, this flare was mild and required a temporary increase in analgesia. The flare usually subsided within 24–48 h. Two patients entered the trial, but were subsequently excluded due to being on cyproterone acetate at the start of the study. One of these patients

**Figure 1** Effect of 4-OHA (500 mg i.m. weekly) on (**a**) serum oestradiol in pmol l$^{-1}$ (**b**), serum testosterone in nmol l$^{-1}$, (**c**), serum 5 a dihydrotestosterone in nmol l$^{-1}$ (**d**), serum androstenedione in nmols l$^{-1}$ (s.d.). Mean values shown with 95% confidence limit bars.

experienced a flare. The three patients in whom the flare was severe, required a marked increase in analgesia and 1 required further palliative radiotherapy.

The endocrine studies did not indicate any correlation between tumour flare and oestradiol suppression or changes in androgen levels during treatment with 4-OHA.

*Nausea and vomiting* Two patients experienced nausea and vomiting on commencing 4-OHA which was treated by conventional anti-emetics. However most patients experienced some degree of nausea prior to treatment, presumable related to narcotic analgesia use.

*Urticaria* One patient experienced mild urticaria soon after commencing 4-OHA. This responded to anti-histamines.

*Vaso-vagal episode* One patient collapsed immediately after a 4-OHA injection. He rapidly recovered. This was thought to be a vaso-vagal episode. This patient discontinued 4-OHA treatment.

### Discussion

The management of patients with advanced prostatic cancer who have failed first line endocrine manoeuvres, such as androgen deprivation, is difficult and is generally aimed at the palliation of symptoms, usually pain. The patients reported in this study all had heavily pre-treated end stage advanced prostate cancer. The quality of subjective response, in the form of increased performance and reduced pain relief, was impressive. The absence of objective responses is not surprising in this type of patient with widespread skeletal metastatic tumour burden.

The tumour flare observed in a high proportion of the patients in this study remains unexplained. The majority of patients demonstrated a fall in E2 during treatment but flare occurred in patients who did not demonstrate a fall in E2. 4-OHA is known to have weak androgenic properties (Brodie *et al.*, 1977) and this may be responsible for the flare. The endocrine results indicate that, in some patients, small increases in T, DHT and A occurred on treatment with 4-OHA. The mechanism of this increase in androgen levels is unknown. No such changes have been noted in post-menopausal female patients with breast cancer (Dowsett *et al.*, 1989) but minor increases were noted in male volunteers with intact gonadal function treated with 4-OHA orally (Dowsett & Lloyd, 1990). In this latter study it was postulated that a competition for catabolic routes of metabolism between 4-OHA and endogenous androgens may be the cause of the increase. There did not, however, appear to be any correlation with changes in androgens and those patients who experienced tumour flare. Cyproterone acetate did not influence the development or course of the flare. It is possible that one or more of the metabolites of 4-OHA are androgenic and responsible for the flare. 4-hydroxytestosterone is a know metabolite in rhesus monkeys treated with 4-OHA (Brodie *et al.*, 1981*b*) but has not been demonstrated in humans. We examined the urine samples of two patients who experienced a tumour flare on 4-OHA by mass spectrometry and HPLC. 4-OHT was not detected (Poon *et al.*, unpublished data). 4-OHT has been shown to bind strongly to the rat androgen receptor (Houghton *et al.*, unpublished data) but whether it is acting as agonist or antagonist is not clear. It is possible that oestradiol suppression reduces the androgen receptor levels or interferes with their response to androgens and 4-OHT/OHA. This may account for the flare subsiding within 24–48 h.

The possibility that aromatase inhibitors may be of benefit in patients with advanced prostate cancer stems from observations made during treatment of such patients with Ag. Originally, it was thought that Ag was acting by reducing adrenal androgens, a 'medical adrenalectomy'. However, it was subsequently shown that any beneficial effects of Ag was not due to this action (Dowsett *et al.*, 1988). Ag is a potent aromatase inhibitor and therefore it seemed important to test a more selective aromatase inhibitor without the central nervous system side effects of AG. 4-OHA is a steroidal aromatase inhibitor which has been extensively investigated in the treatment of women with advanced breast cancer (Goss *et al.*, 1984). Much data have been accrued concerning the effects of such inhibitors in the female but little is known concerning their effects in man. Our preliminary study (Shearer *et al.*, 1990) indicated that oestrogen suppression occurred during treament with 4-OHA and it was considered that this may be responsible for the benefit observed. Oestrogens have attracted considerable interest in connection with prostatic biology particularly in the pathogenesis of benign prostatic hyperplasia (BPH) (Henderson et al., 1986). Several lines of evidence suggest that oestrogens may be involved in the development of BPH (Matzkin et al., 1991). Whether oestrogens play a role in the development and progression of prostatic carcinoma remains conjectural. We have conducted parallel laboratory studies which have shown that the aromatase enzyme does not appear to be present in either benign or malignant prostatic tissue *in vitro* (Davies et al., 1989). It has been reported that 4-OHA inhibits human prostatic 5 a reductase (Zoppi et al., 1988) enzyme activity. However, we have found that this inhibition is very weak in human benign and malignant prostatic tissue *in vitro* (Davies et al., 1989). This clinical study found no relationship between subjective response and oestradiol suppression. The mechanism of action of 4-OHA therefore remains unclear.

The beneficial effects of 4-OHA may be due to an action unrelated to its aromatase inhibitory activity. Inhibition of prostaglandin synthetase activity as a possible mode of action by AG in patients with prostate cancer (Harris et al., 1983) or a central action (Santen et al., 1981) has been suggested. Whether this is the case in patients treated with 4-OHA is unsubstantiated.

In conclusion, we have found 4-OHA to be effective in palliating patients with advanced prostate cancer who have failed all other palliative measures. The mechanism of action of 4-OHA in such patients may be by suppressing oestradiol, altering oestrogen related bone metabolism and hence reducing metastatic activity. Further work in this area would appear worthwhile and may lead to further understanding of the effects of oestrogens on prostatic biology and on metastatic prostate cancer.

4-OHA was supplied by Ciba-Geigy Pharmaceuticals, Horsham. The support of Dr S. Hughes and Mr N. Adams at Ciba-Geigy Pharmaceuticals, Horsham is gratefully acknowledged. JHD was supported by a grant from Ciba-Geigy Pharmaceuticals.

### References

BRODIE, A.M.H., SCHWARZEL, W.C., SCHAIKH, A.A. & BRODIE, H.J. (1977). The effects of an aromatase inhibitor, 4-hydroxy-4-androstene-3,17-dione, on oestrogen-dependant processes in reproduction and breast cancer. *Endocrinology*, **100**, 1684.

BRODIE, A.M.H., GARRET, W.M., HENDRICKSON, J.R., TSAI-MORRIS, C.H., MARCOTTE, P.A. & ROBINSON, C.H. (1981*a*). Inactivation of aromatase in-vitro by 4-hydroxy-4-androstene-3,17-dione and 4-acetoxy-4-androstene-3-17-dione and sustained effects *in vivo*. *Steroids*, **38**, 693.

BRODIE, A.M.H., ROMANOFF, L.P. & WILLIAMS, K.I.H. (9181*b*). Metbolism of the aromatase inhibitor 4-OHA by male rhesus monkeys. *J. Steroid Biochem.*, **14**, 693.

COOMBES, R.C., GOSS, P., DOWSETT, M., GAZET, J.-C. & BRODIE, A. (1984). 4-hydroxyandrostenedione in treatment of menopausal patients with advanced breast cancer. *Lancet*, **xii**, 1237.

DAVIES, J.H., ROWLANDS, M.G., DOWSETT, M., COOMBES, R.C. & SHEARER, R.J. (1989). *In vitro* studies of the effects of 4-OHA on androgen metabolism in human benign and malignant prostatic tissues. *Proceedings of the AACR*, **30**, 303(1204).

DOWSETT, M., GOSS, P.E., POWLES, T.J. & 4 others (1987). Use of the aromatase inhibitor 4-hydroxyandrostenedione in post-menopausal breast cancer: Optimization of therapeutic dose and route. *Cancer Res.*, **47**, 1957.

DOWSETT, M., SHEARER, R.J., PONDER, B.A.J. & MALONE, P. (1988). The effects of aminoglutethimide and hydrocortisone, alone and combined, on androgen levels in post-orchiectomy prostatic cancer patients. *Br. J. Cancer*, **57**, 190.

DOWSETT, M., CUNNINGHAM, D.C., STEIN, R. & others (1989). Dose related endocrine effects and pharmacokinetics of oral and intramuscular 4-hydroxyandrostenedione in postmenopausal breast cancer patients. *Cancer Res.*, **49**, 1306.

DOWSETT, M. & LLOYD, P. (1990). Comparison of the pharmacokinetics and pharmacodynamics of unformulated and formulated 4-hydroxyandrostenedione taken orally by healthy men. *Cancer Chemother. Pharmacol.*, **27**, 67.

HARRIS, A.L., MITCHELL, M.D., SMITH, I.E. & POWLES, T.J. (1983). Suppression of plasma 6-keto-prostatglandin Fla and 13,14-dihydro-15-keto-prostaglandin F2a by aminoglutethimide in advanced breast cancer. *Br. J. Cancer*, **48**, 595.

HENDERSON, D., HABENICHT, U.-F., NISHINO, Y., KERB, B. & EL ETREBY, M.F. (1986). Aromatase inhibitors and benign prostatic hypertrophy. *J. Steroid Biochem.*, **25**, 867.

LANCET EDITORIAL (1980). Cancer of the prostate. *Lancet*, **ii**, 1009.

MATZKIN, H. & BRAF, Z. (1991). Endocrine treatment of benign prostatic hypertrophy: Current concepts. *Urology*, **37**, 1.

PARKER, M.C., COOK, A., RIDDLE, P.R., FRYATT, I., O'SULLIVAN, J.P. & SHEARER, R.J. (1985). Is delayed treatment justified in carcinoma of the prostate? *Br. J. Urol.*, **57**, 724.

PONDER, B.A.J., SHEARER, R.J., POCOCK, R.D. & 4 others (1984). Response to aminoglutethimide and cortisone acetate in advanced prostate cancer. *Br. J. Cancer*, **50**, 757.

ROBINSON, M.R.G. (1980). Aminoglutethimide: Medical adrenalectomy in the management of carcinoma of the prostate. A review after 6 years. *Br. J. Urol.*, **52**, 328.

SANTEN, R.J., SAMOJLIK, E. & WELLS, T.J. (1981). Aminoglutethimide product profile. In *A Comprehensive Guide to the Therapeutic use of Aminoglutethimide*, Santen, R.J., Henderson, I.C. (eds). p 101. Karger: Basel.

SHEARER, R.J., DAVIES, J.H., DOWSETT, M. & 4 others (1990). Aromatase inhibition in advanced prostatic cancer: preliminary communication. *Br. J. Cancer*, **62**, 275.

WORGUL, T.J., SANTEN, R.J., SAMOJILK, E., VELDHUIS, J.D., LIPTON, A. & HARVEY, H.A. (1983). Clinical and biochemical effects of aminoglutethimide in the treatment of advanced prostate cancer. *J. Urol.*, **129**, 51.

ZOPPI, S., COCCONI, M., LECHUGA, M.J., MESSI, E., ZANISI, M. & MOTTA, M. (1988). Antihormonal activities of 5 alpha reductase and aromatase inhibitors. *J. Steroid Biochem.*, **31**, 4B, 677.